# Modeling Malaria Infection and Immunity against Variant Surface Antigens in Príncipe Island, West Africa

**DOI:** 10.1371/journal.pone.0088110

**Published:** 2014-02-10

**Authors:** Cátia Bandeiras, Maria Jesus Trovoada, Lígia A. Gonçalves, Cláudio R. F. Marinho, Louise Turner, Lars Hviid, Carlos Penha-Gonçalves, M. Gabriela M. Gomes

**Affiliations:** 1 Instituto Gulbenkian de Ciência, Oeiras, Portugal; 2 Centre for Medical Parasitology, University of Copenhagen and Copenhagen University Hospital (Rigshospitalet), Copenhagen, Denmark; Universidade Federal de Minas Gerais, Brazil

## Abstract

After remarkable success of vector control campaigns worldwide, concerns about loss of immunity against *Plasmodium falciparum* due to lack of exposure to the parasite are relevant since an increase of severe cases in less immune individuals is expected. We present a mathematical model to investigate the impact of reducing exposure to the parasite on the immune repertoire against *P. falciparum* erythrocyte membrane protein 1 (PfEMP1) variants. The model was parameterized with data from Príncipe Island, West Africa, and applied to simulate two alternative transmission scenarios: one where control measures are continued to eventually drive the system to elimination; and another where the effort is interrupted after 6 years of its initiation and the system returns to the initial transmission potential. Population dynamics of parasite prevalence predict that in a few years infection levels return to the pre-control values, while the re-acquisition of the immune repertoire against PfEMP1 is slower, creating a window for increased severity. The model illustrates the consequences of loss of immune repertoire against PfEMP1 in a given setting and can be applied to other regions where similar data may be available.

## Introduction

Remarkable success of malaria control campaigns has been achieved in the last decade, with a 25% decrease in worldwide deaths [1]. Many of the control campaigns rely on vector control interventions that have been especially effective in reducing the transmission of *Plasmodium falciparum*, the most deadly human malaria parasite [2]–[3]. Transmission reduction comes with the side effect of promoting loss of naturally acquired immunity against parasite antigens that are important in controlling the progression of the parasite life cycle in the host and preventing complicated disease outcomes [4]. With many obstacles arising for *P. falciparum* elimination, such as insecticide and drug resistance, high genetic diversity and breakdown of control campaigns [5]–[7], transmission might re-emerge, with infections causing more severe disease due to declining immunity levels [4].

The *P. falciparum* erythrocyte membrane protein 1 (PfEMP1) family is very important both for the pathogenesis of falciparum malaria and for naturally acquired immunity to the disease [8]. This protein is responsible for the cytoadherence of infected erythrocytes to vascular endothelial receptors and plays an important role in malaria pathogenesis by modifying the microcirculation and allowing parasites to escape clearance by the spleen [9]. PfEMP1 is a variable surface antigen (VSA) of *P. falciparum* and is a major factor of immune evasion by the parasite since it has the ability of switching expression amongst different variants, a process known as antigenic switching [10]. The presentation of variants to the host is hierarchical, in the sense that dominant variants are more cytoadherent and more likely to cause severe disease, being predominantly expressed in naïve hosts. As hosts acquire immunity, less dominant variants are expressed [11]–[13]. Due to the considerable intraclonal and interclonal variability of genes encoding for this protein (*var* genes, about 60 per haploid genome and with fast recombination) [14], immunity against PfEMP1 is practically never fully acquired. However, individuals from endemic areas are able to maintain a broad antibody repertoire due to persistent exposure to the parasite [15]. Upon reducing transmission, there is concern that waning serological immunity to PfEMP1 antigenic variants may render individuals more vulnerable to disease.

Mathematical modeling is a useful tool in the assessment of transmission scenarios and control measures [16]. It has also been used to relate transmission with measures of serological markers [17], to simulate the dynamics of within-host acquisition of PfEMP1 variants and its impact on the life cycle of the parasite [18]–[20], and to study evolutionary mechanisms acting on *var* genes at the population level [12]; [21]. In this work, we link transmission scenarios in a setting undergoing control measures towards elimination with the breadth of the immune repertoire against PfEMP1. The dataset used was collected in Príncipe Island, the smallest island of São Tomé and Príncipe, an archipelago in West Africa. Príncipe has been classified as a meso/ hyperendemic malaria region with perennial transmission in most areas. Since 2004, control measures have been very successful, with the island having low and stable transmission since 2007 [22]. However, measures of transmission and identification of malaria carriers were mainly based on optical microscopy diagnostics. We use, along with serological data for a subset of intraclonal variability of PfEMP1, infection data obtained by polymerase chain reaction (PCR), a technique that detects more efficiently the asymptomatic cases that constitute a reservoir for transmission [23].

## Materials and Methods

### Informed consent and ethical permits

Ethical permit to conduct this study was granted by the Ministry of Health of S. Tomé and Príncipe in the scope of a collaborative protocol between the Fundação Calouste Gulbenkian and the S. Tomé e Príncipe government on Malaria research. Written informed consent was obtained from every participant. In case of children participants, written consent was obtained from the guardians.

### Sample collection

In 2005 a population-based study was conducted, covering most villages in the Príncipe Island. Blood samples were collected from apparently healthy participants without any prior selection or restrictions on participation. The age distribution of the sampled individuals ranged from 1 month to 85 years of age. For each participant, a malaria clinical and epidemiological enquiry was filled in.

### Malaria carrier detection

To detect parasite DNA in peripheral blood, DNA was extracted using 96 Qiamp Blood kit. A nested PCR amplification assay was used for the detection of the main four human malaria species as described in [24].

### Anti-parasite antibodies

The anti-*var* antibody profiling technique has been used to identify naturally acquired immunity to *P. falciparum* [13] and can be summarized as follows: Forty-five recombinant proteins representing domains present in different PfEMP1s from the 3D7 clone were expressed. The domains were chosen to represent PfEMP1s belonging to different groupings (groups A, B/A, B or C) comprising domains of different types and located in different regions of the proteins. For a more detailed description of the tested variants see Table S1. Carboxylated luminex microspheres were covalently coupled with the different PfEMP1 domains according to the manufacturer’s protocol. Each protein was coupled to a particular fluorescence-coded microsphere type. To determine domain-specific IgG levels in the plasma, lyophilized microspheres were reconstituted immediately prior to use. Frozen plasma samples were thawed at room temperature, diluted by 1∶80 in ABE buffer and 50 µl aliquots were added to the microsphere wells. After incubation, Biotinylated anti-human IgG antibody was added to the microspheres. This was followed by streptavidin-phycoerythrin. Finally, the microspheres were re-suspended in and analyzed on a Luminex 100 IS instrument set to read a minimum of 100 microspheres per microsphere region. Antibody levels for each domain were expressed as median fluorescent intensity (MFI). We used a seronegativity cut-off value based on the reactivity measured in plasma from unexposed control donors.

### Data processing and management

All data were double entered and checked in Microsoft Excel spreadsheets. Initial conditions for the simulations were estimated from the 2005 survey data. Individuals with incomplete fields such as age and parasitemia, as well as with insufficient serum for serological analysis, were not considered for further analysis. After these adjustments, 535 samples with age, parasitological and serological information were obtained. To avoid possible immunity confounders from infections with other *Plasmodium* parasites, a subset with only uninfected and *P. falciparum* infected individuals (including mixed infections) was obtained, consisting of 517 individuals. To remove the effect of possible maternally-acquired immunity, individuals aged less than 1 year at the time of the survey were removed from the analysis. For each individual, the number of acquired antibody specificities and variants was calculated. To test whether the acquisition of antibodies for different epitopes of the same PfEMP1 variant is related, where applicable, Spearman’s correlation test was applied.

### Models of infection and immunity dynamics

For the infection dynamics, we adopted a susceptible-infected-susceptible (SIS) framework derived from the basic model of Ross [25]. The model is formulated as a system of partial differential equations on age and time [26], and the endemic equilibrium that this describes was fitted to the prevalence data. The data was organized by structuring the 2005 sample population into 9 age groups and, initially, two model parameters were estimated: the transmission coefficient (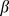) and the clearance rate (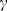). It was assumed that, before the 2005 survey, the population was in endemic equilibrium to allow parameter estimation using the age prevalence data from the initial survey. Demography was included in the model by a birth and death rate (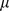) considered as the inverse of the mean life expectancy in São Tomé and Príncipe in 2005 (63.9 years) [27]. For the proposed model, the basic reproduction number (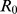) is approximately the ratio of the transmission coefficient by the clearance rate, and we opted to estimate 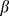 and 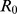 in the first instance, and from these calculate 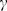. This approach minimized correlation between the parameters and improved confidence on the estimates. To obtain parameter estimates using a Bayesian framework, Markov Chain Monte Carlo (MCMC) methods were implemented using the Metropolis-Hastings algorithm. The priors for the two parameters were uniformly distributed, ranging from -5 to 3 in log_e_ scale. The model likelihood is based on the binomial distribution of the proportion of infected individuals. Two chains were run in parallel starting from overdispersed initial values to ensure convergence of the chain irrespectively of the starting value. The chains were run for 45,000 iterations, with a burn-in period of 100 iterations and a thinning factor of 45, resulting in 1000 posterior samples for parameter inference. Stationarity of each chain was assessed using the Heidelberg and Welch test and convergence of the overdispersed chains to the same stationary distribution was confirmed by applying the Gelman-Rubin diagnostic. Time simulations of the population-wide infected proportions were run by implementing an intervention that decreases the transmission coefficient to one half. Scenarios were projected by simulating the model until 2024, under two alternative assumptions regarding the transmission coefficient: (i) remaining at the reduced level achieved by the intervention; (ii) returning to the original level 6 years after the start of the intervention.

**Figure 1:**
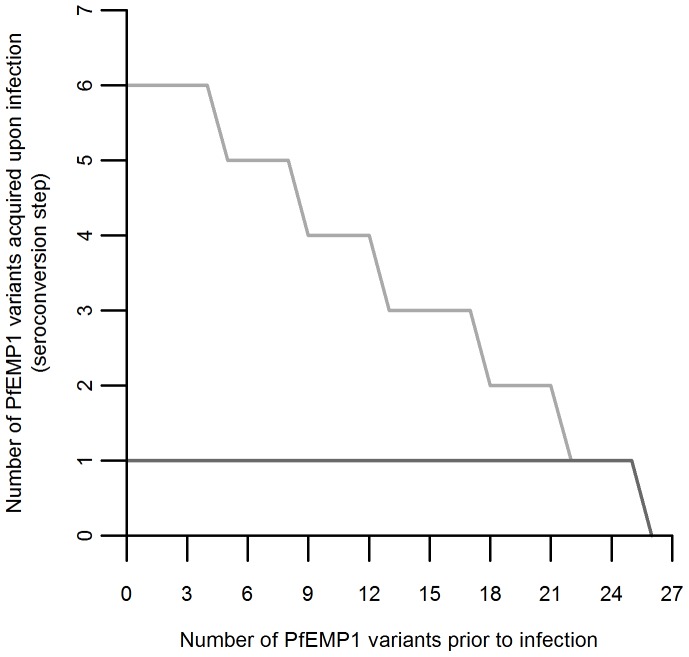
Seroconversion step with respect to the number of PfEMP1 variants in the immune repertoire prior to infection. Dark gray: Situation with no antigenic switching implied, acquisition of one variant per infection event. Light gray: Situation with antigenic switching, with the number of variants acquired per infection varying with the number of variants the individual has prior to infection. Example assuming a seroconversion step of 6 variants in a completely seronegative individual.

Acquisition and loss of antibodies against PfEMP1 variants expressed by specific *var* genes was modeled through a counting process of the number of PfEMP1 variants an individual has acquired immunity to. The framework hereby presented extends reverse catalytic models used to study seroconversion and seroreversion to a given antigen [17] by applying queuing theory as in previous formulations of superinfection [28]. Detection of antibodies was focused on a set of 26 variants, thus a system of 27 partial differential equations was developed to describe the dynamics of acquisition and loss of immunity against PfEMP1 variants. Each state describes the number of variants a person has immunity to. Seropositivity was described as having a mean fluorescent intensity (MFI) above a cut-off value obtained from a cohort of naïve individuals (see data description). Preserving the aforementioned assumption of endemic equilibrium, the parameters of the model, seroconversion (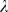) and seroreversion (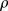) rates, were estimated based on the 2005 data by grouping the 27 compartments into 3 categories: seronegative individuals; individuals with immunity to 1–10 variants; and individuals with immunity to 11 or more variants. Initially, the model represented acquisition of immunity in unitary steps (i.e. one variant per infection event) at rates independent of the prior number of variants. In order to take into account the possibility of an infected individual being exposed to more than one variant during a single infection due to the process of antigenic switching, the model was extended to allow for the acquisition of immunity to more than one variant during a single infection. To the number of variants acquired in the course of a given infection we call the seroconversion step. The seroconversion step is assumed to decrease linearly with the number of variants recognized by the immune system prior to each specific infection. Figure 1 illustrates how this decrease is approximated by a step function since only integer values are permissible.

Regarding the loss of antibodies, the rate of seroreversion is the same for all the states and independent for each variant. Since the factors that modulate seroconversion in each state are fixed, two parameters remain to be estimated (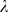 and 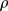). Models with different initial seroconversion steps were compared with the unitary step model by comparing the Akaike Information Criterion corrected for small sample size (AICc). The model with the minimum value of AICc is considered the best fitting model and will be used for time simulations. For parameter estimation of the seroconversion rate and the seroreversion/seroconversion ratio for each model, to avoid correlation issues, MCMC chains were run in a similar manner as described for the transmission model. Uniform priors were used for both parameters and the likelihood had a multinomial distribution. Chains were run for 160,000 iterations with a burn-in period of 2000 iterations and 1000 iterates kept for parameter inference. The applied convergence tests were the same as for the transmission model and 95% credible intervals were calculated. Time simulations of the PfEMP1 immune repertoires were performed through the vector control campaign by applying to a seroconversion reduction factor calculated from the imposed reduction in the transmission coefficient.

### Model equations

**1) SIS model.** The partial differential equation (PDE) system in age and time domains that was used to model transmission is written as follows: 


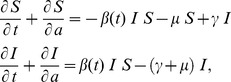
(1)where 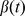 is implemented in a stepwise manner to mimic the intervention:
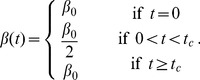
(2)


In (2), 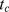 represents the time of intervention breakdown, here considered as 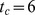 years. The case where the intervention is maintained indefinitely is formalized as 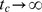. The *per capita* rate of infection (force of infection) is 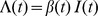, and the boundary conditions for the system at age *a*  =  0 are:
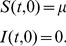
(3)


For ease of visual representation of the obtained results and fitting, the proportions were normalized with respect to the approximated distribution of population over age
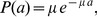
(4)with the fittings being performed using the normalized differential equations and adjusting to the proportions of infected individuals in each age group.

The non-trivial equilibrium solution for the infected compartment of the system in (1) is:



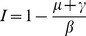
(5).

From (5), we derive the basic reproduction number, 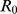, as:



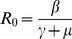
(6).

**2) PfEMP1 immunity model.** First we describe the model for acquisition of one PfEMP1 variant per event and then extend it to accommodate the acquisition of immunity to multiple variants.

Let 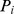 represent the state “seropositive to *i* variants”. Therefore, 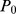 represents the seronegative individuals, that is, those who have not been exposed to the parasite or have lost immunity to the set of variants previously acquired. At the other end, 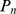 represents the individuals that have been exposed and retain immunity to all the *n* variants considered in this representative set (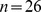 as previously mentioned). The model is represented as follows:
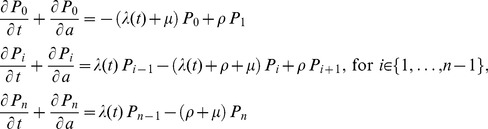
(7)


with boundary conditions at age *a*  =  0, assuming no maternally-acquired immunity, given by:
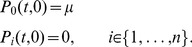
(8)


The fitting procedure also involved the calibration of the normalized differential equation values in the age domain with the proportions of individuals in each of the three categories defined for estimation, specifically: 0 variants; 1–10 variants; more than 11 variants. The seroconversion rates vary over time proportionally with the variation of the force of infection estimated by the SIS model:



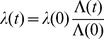
(9).

For the model concerning a seroconversion step that decreases with the number of variants in the immune repertoire prior to infection, we developed a stepwise framework that approximates a linear dependence. Consider the seroconversion step when an infection occurs in an individual on the state 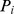 to be given by:



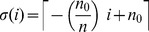
(10)where 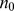 represents the seroconversion step in completely seronegative persons, that is, when *i*  =  0. To know the final state, 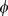, after an event of seroconversion we sum the initial state to the seroconversion step:



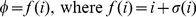
(11)


Seroconversion events are then represented as transitions from initial states 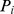 to final states 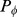 with rates determined by the basic rule just described. Seroreversions are more simply implemented as transition from 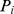 to 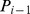 at fixed rates for all individuals regardless of their age/immune status.

According to the described seroconversion mechanism, the set of indices, *i*, that lead to a given final state, 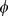, may contain zero, one or two elements as given by the inverse operation 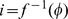. The full system is then represented by the following set of equations:
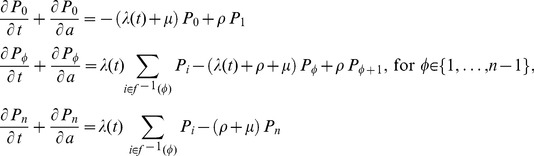
(12)while an expanded representation for the specific scenario 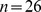 and initial seroconversion step of 6 (illustrated in Figure 1) is provided in Text S1.

The mean number of antibodies at age *a* is calculated from the estimated 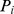
’s. Finally, the seroconversion step, which we have so far represented in terms of the number of variants prior to infection, can also be represented in terms of age at infection by calculating the mean number of antibodies by age, 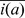, and applying the following transformation:



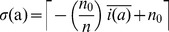
(13)


### Model implementation and parameter estimation

For parameter estimation purposes, equilibrium in time was assumed and the resulting models were implemented as ordinary differential equations (ODEs) in the age domain and solved numerically using the “deSolve” package of R v2.14.1. To select the best-fitting models for the PfEMP1 variant dynamics, maximum likelihood estimates based on a multinomial error distribution were performed and compared using AICc with functions from the “bbmle” package in R. MCMC parameter estimation procedures were also implemented in R and evaluated with several diagnostic procedures through functions of the packages “MCMCpack” and “coda”. For time simulations, the system of PDEs was implemented and solved in MATLAB R2011a using the “pdepe” routine, considering the solutions of the age-dependent ODEs for each age as the initial conditions for time simulation.

## Results

### Parasite surveys

Using the 535 samples of the serological and parasitological survey in 2005, the total prevalence of malaria infections is 30.28% (162/535). The relative percentage of infections is shown in Table 1. When subsetting to a group with only *P. falciparum* infections (including mixed infections) and uninfected individuals, the prevalence of *P. falciparum* infections is 27.85% (144/517).

**Table 1:** Global prevalence of infection in 2005 in Príncipe Island and relative prevalence of each type of infection.

Infection type	Prevalence	Relative prevalence
*P. falciparum*	23.93% (128/535)	79.01% (128/162)
*P. vivax*	1.31% (7/535)	4.32% (7/162)
*P. ovale*	0.75% (4/535)	2.47% (4/162)
*P. malariae*	1.12% (6/535)	3.70% (6/162)
Mixed	3.18% (17/535)	10.49% (17/162)

### Relationship between antibodies for the same PfEMP1 variant

Out of the 26 recombinant PfEMP1 in the dataset, 11 had more than one epitope represented. In order to know if an increase in the levels of one epitope led to an increase in the levels of other epitopes of the same variants, Spearman’s correlation was computed for these variants. For 8 of the 11 variants, the Spearman correlation coefficient was above 0.7, indicating a high positive correlation between the levels of antibodies for epitopes of the same variant. All the responses were positively correlated, confirming that acquisition of antibodies for epitopes of the same variant is not independent and laying the foundation for a model based on recognition of variants instead of presence of antibodies against individual epitopes.

### Transmission model and simulations

**Figure 2:**
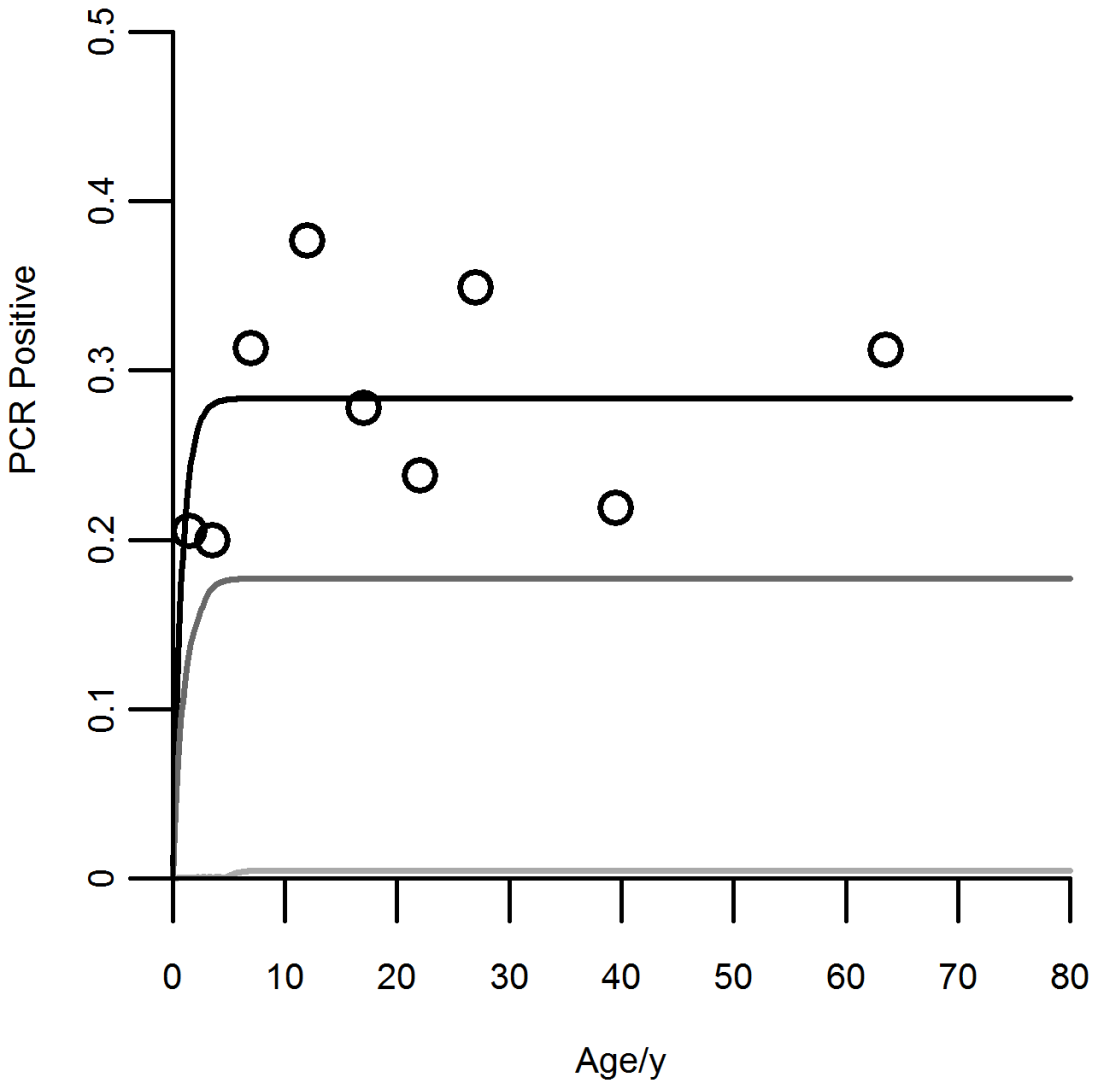
Infection data by age in 2005, SIS model (1) fit and projected intervention impacts. True proportions per age group are represented by black circles. Black curve: SIS model with the best fit parameters in 2005. Dark gray curve: Prediction of the infected proportions by age in 2008 (2.5 years after the beginning of the intervention). Light gray curve: Prediction of the infected proportions by age in 2010 (5 years after the beginning of the intervention).

Due to high correlation of the estimates for parameters 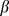 and 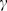 of the SIS model (1), we opted to estimate 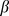 and 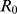 and calculated the value of 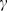 using the equilibrium derivation in (5) and (6). The estimated values for 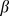 and 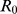, as well as for the dependent parameters 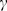 and 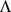, and the 95% credible intervals, are present in Table 2. The best fitting model simulation with respect to the original infection data is shown in Figure 2, as well as time projections for the following 2.5 and 5 years, assuming an intervention where the transmission coefficient 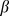 is reduced to one half of the initial value.

**Table 2:** Mean and median values with 95% credible intervals for the distributions of estimated parameters for the SIS model (1).

Parameter	Mean estimate	Median estimate	95% CI
*β*	3.249	1.236	0.3553–16.606
*γ*	2.3280	0.8695	0.2339–11.8092
*R*_0_	1.424	1.419	1.3327–1.537
Λ	0.9211	0.3627	0.1170–4.5552

The distribution of 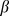 is skewed to low positive values but the credible intervals are broad due to the presence of outliers. These outliers may be related to heterogeneous exposure, particularly focal areas of high exposure. Due to the high correlation of 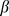 and 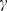, the high variability in the estimates does not translate into a high variability of the basic reproduction number (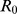), estimated to be slightly above 1, as of 2005. The estimated clearance rate 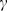 in 2005 is 2.3280 year^−1^ according to the mean statistic (duration of infection of about 5 months) and 0.8695 year^−1^ according to the median (duration of infection of about 1 year). These values are compatible with estimates obtained by fitting a panel of refined models to data from more than 90 communities [29]. Moreover, the broad credible intervals accommodate scenarios of faster clearance in the case of clinical infections undergoing treatment, as suggested by previously models [30]–[31].

**Figure 3:**
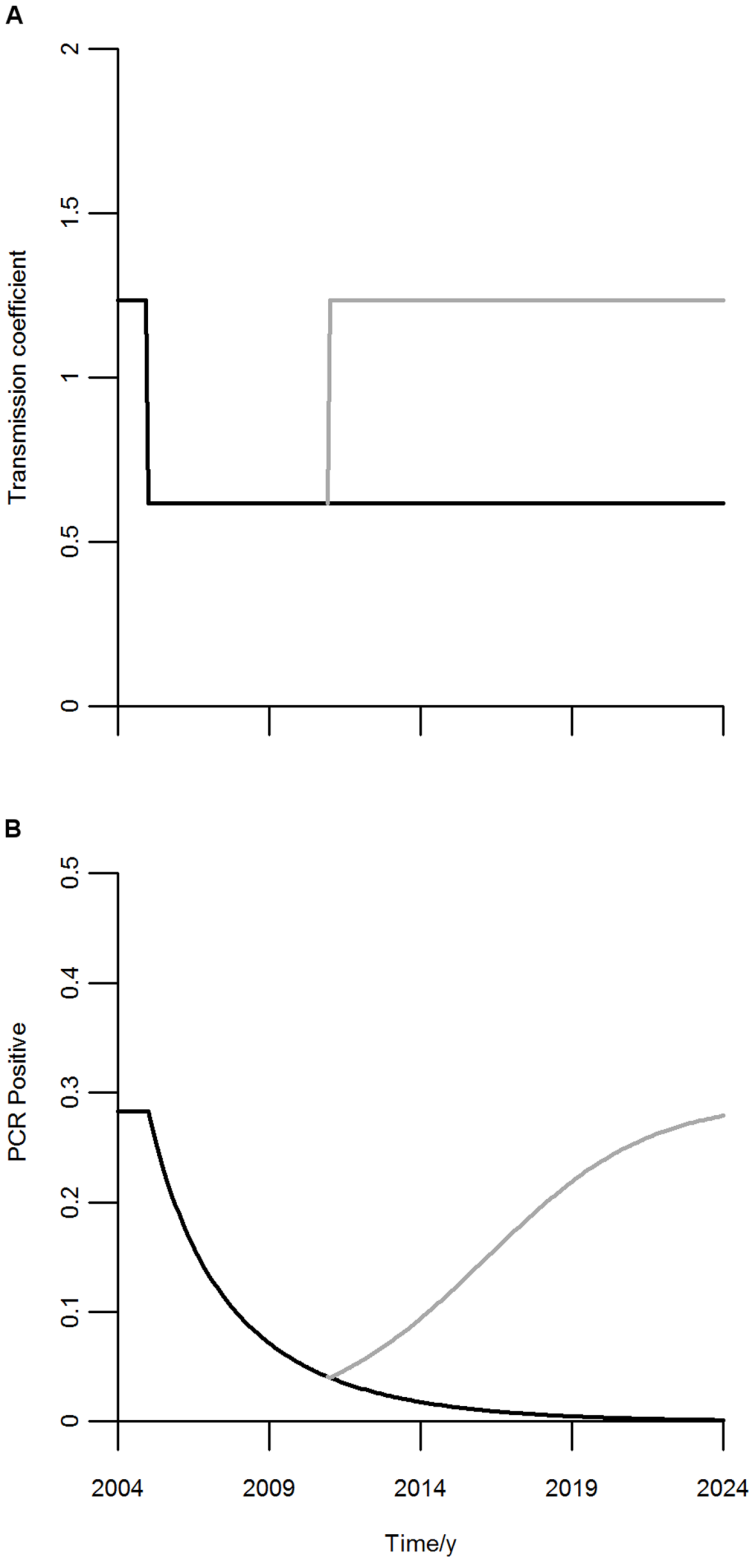
Implementation of transmission changes and associated infected dynamics. A: Black: Simulation of an intervention that reduces the transmission coefficient to one half of the pre-intervention equilibrium value. Gray: The same intervention but with relapse to the pre-intervention value in 2011 (6 years after the intervention started). B: Curves generated by model (1). Black: Infected proportions over time when the transmission coefficient is reduced to one half, indefinitely. Gray: Infected proportions over time when the transmission coefficient is reduced to one half, returning to the initial value in 2011.

Using the median values for the obtained estimates, since these are more robust measures of the characteristics of the population, we performed time simulations of the proportion of infected individuals. Simulations were implemented by inducing a decrease in the transmission coefficient to one half beginning in 2005. After this initial period two scenarios were simulated: one where the intervention effort continues indefinitely; and another where the intervention is halted in 2011 (6 years after its start) and the transmission potential returns to the original level. The values of the transmission coefficient and the simulated prevalence results are shown in Figure 3.

With the reduced transmission coefficient, the proportion of infected individuals will decrease gradually and, therefore, the force of infection will decrease as well. If the simulated control measures were kept with the same effort indefinitely, the level of infected individuals would decrease to very low values (circa 1% of infected individuals) after 20 years, eliciting good prospects for elimination. After only 5 years of effective intervention measures (Figures 2 and 3B), the prevalence of infection would fall below 5%, being very low for every age group. However, if control measures are interrupted and the transmission coefficient returns to the pre-intervention value, within a decade after the intervention breakdown the population levels of infected individuals return to levels near the initial proportion of c.a. 28%. In this simulation there is no distinction between clinical and asymptomatic, or between infectious and non-infectious cases, but it is plausible that loss of population immunity due to reduced exposure to the parasite could result in increased severity and infectivity in case of re-emergence.

### PfEMP1 immunity model and simulations

The results of the fittings by maximum likelihood methods using a multinomial error for the immunity model (13) with different seroconversion steps are shown in Table 3, as well as the respective AICc values.

**Table 3:** Comparison between implementations of the PfEMP1 model (13) with different seroconversion steps.

Initial step (*nvars*)	Λ	Ρ	AICc
1	14.2684	14.3189	142.5053
2	2.7416	4.5700	133.6528
3	1.5154	3.3920	121.1597
4	1.0570	2.9597	109.3547
5	0.7656	2.6473	99.1733
6	0.6347	2.5230	97.1156
7	0.5300	2.4491	98.4256
8	0.4863	2.5446	104.1059
9	0.4596	2.6981	111.6555
10	0.4313	2.7984	120.1354
11	0.4164	3.0210	138.1856
12	0.4387	3.5201	157.8700
13	0.4846	4.1849	175.283

Evaluation of the best initial step is performed by computing the Akaike Information Criterion with small sample size correction (AICc).

**Figure 4:**
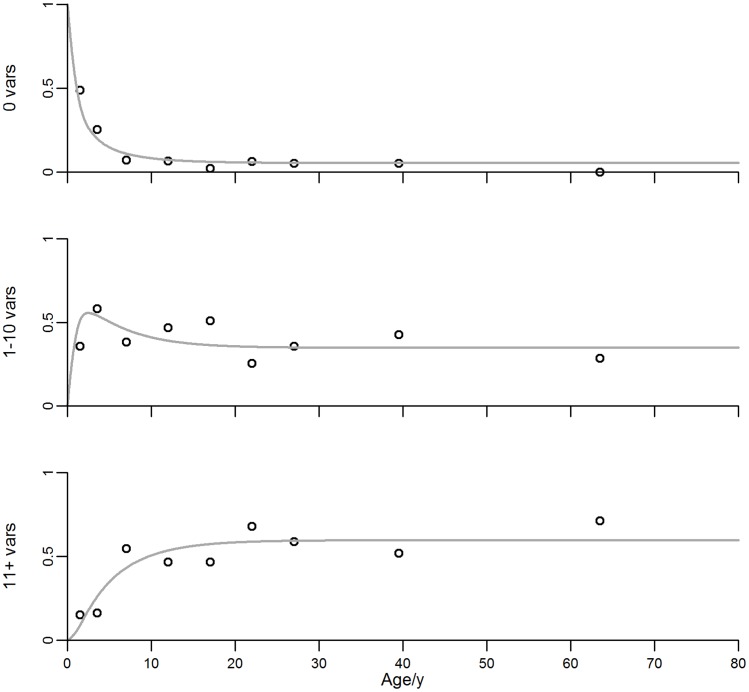
PfEMP1 model (13) with initial seroconversion step of 6 variants and age data in 2005. True proportions per age group are represented by black circles. Gray curves: Estimated proportions by age for each category. Top: Individuals with no immunity. Middle: Individuals with immunity to 1–10 variants. Bottom: Individuals with immunity to more than 11 variants.

**Figure 5:**
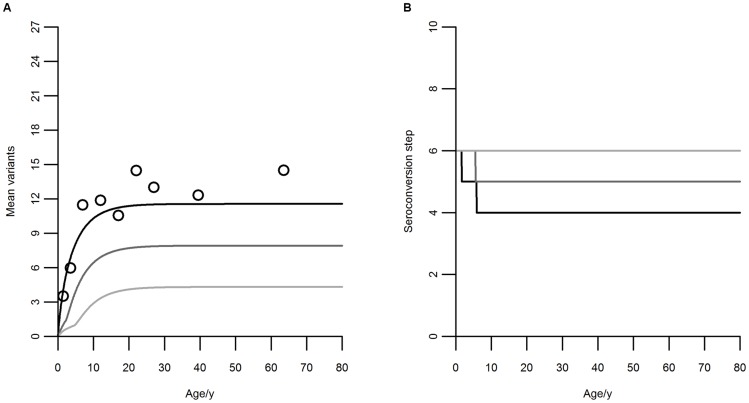
Equilibrium antibody repertoires and seroconversion steps by age with initial step of 6 variants. A: Mean number of PfEMP1 variants by age. Black circles: true means per age group in 2005. Black curve: model (13) fit to 2005 data. Dark gray: projection for 2008 (2.5 years after the beginning of the intervention). Light gray: projection for 2010 (5 years after the beginning of the intervention). B: Seroconversion step by age. Black curve: 2005, from the best-fit model. Dark gray: 2008, projected. Light gray: 2010, projected.

Using this criterion, it is clear that the best fit, corresponding to the smaller value of AICc, is provided by the model with an initial seroconversion step of 6 *var* genes. The parameter estimation results for the model with initial seroconversion step of 6 variants, as well as the respective 95% credible intervals, is shown in Table 4. Graphical representation of the best fitting model against proportions of individuals in each of the antibody repertoire categories can be seen in Figure 4. The mean number of antibody variants and seroconversion steps by age are shown in Figure 5.

**Table 4:** Parameter estimates for the PfEMP1 model (13) with state-dependent seroconversion step and initial step of 6 variants.

Parameter	Mean estimate	Median estimate	95% CI
λ	0.6468	0.6394	0.4988–0.8350
ρ	2.5799	2.5500	1.9250–3.4170

**Figure 6:**
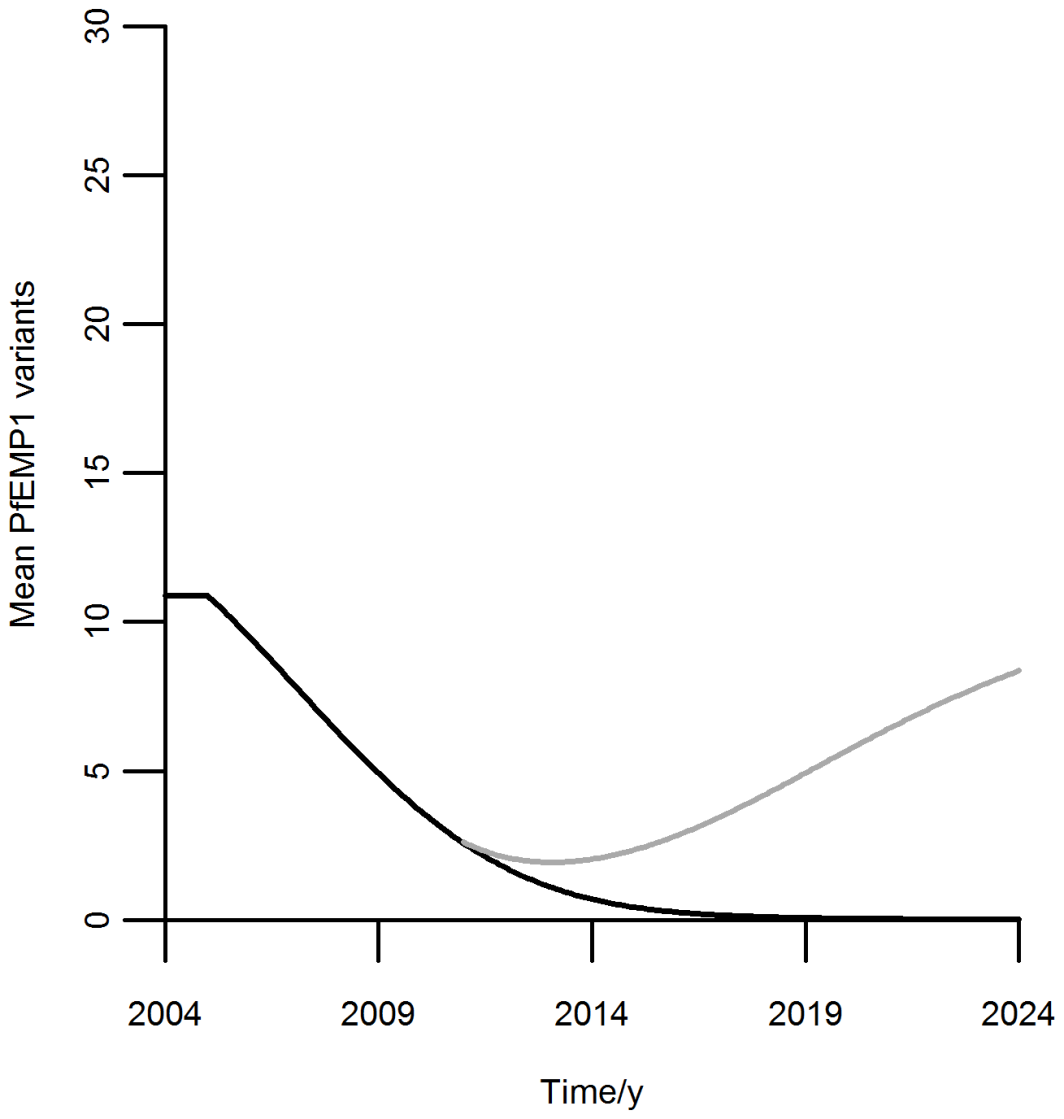
PfEMP1 variants averaged over the population assuming initial seroconversion step of 6 variants. Curves generated by model (13). Black: transmission coefficient is reduced to half of its initial value, until the end of the simulation. Gray: transmission coefficient is reduced to half of its initial value, and relapses back to the initial value in 2011 (6 years after the intervention started).

The impact of transmission changes on the broadness of the immune repertoire of the individuals in the population is shown in Figure 6. The simulations were performed using the same transmission coefficient factors to modulate both infection and seroconversion rates, according to expression (9).

Under the model where up to 6 variants are acquired in a single infection, the loss of antibodies when transmission decreases is considerable within 5 years of intervention, with a considerable proportion of the population being immunologically naïve due to lack of exposure (Figure 5A and 6). This may lead to considerable risk of severe disease in the years after transmission re-emergence, mostly evident in older individuals in highly endemic settings. The potential for seroconversion given a new infection (Figure 5B) increases to the expected value for naïve individuals and, although this facilitates the re-establishment of immunity, the pace of immunity recovery remains slower than that of infection rebound (compare Figures 3B and 6). In fact, the mean number of variants in the population does not return to the pre-intervention values within twenty years of pre-intervention levels of transmission while infection does.

The unitary seroconversion step model (7) predicts a faster decay of immunity and even slower recovery upon transmission rebound. For comparison we refer to supporting information, where Table S2 can be contrasted with Table 4, and Figures S1–S3 with Figures 4, 5, 6, respectively. Although the 2005 data presented here gives better support for the model where up to 6 variants are acquired in a single infection it would be interesting to confront these predictions with follow-up data from the island.

## Discussion

We have parameterized infection and immunity models to represent the endemic state of *P. falciparum* malaria in the Príncipe Island in 2005, and simulated intervention scenarios. One of the strengths of this study is the availability of a detailed dataset relating PfEMP1 antibody repertoires with host age and parasite prevalence. As interventions to reduce transmission in the island have started to gain significance just after the collection of this data, our study reports a unique pre-intervention analysis essential for future intervention assessment. Simulations are consistent with the qualitative expectations that reduced transmission will result in decrease of exposure, narrowing the immune repertoire at the population level and reducing disease protection. More quantitative descriptions can be generated by adjusting these models to similar data collected post-intervention, as well as to provide a more accurate parameterization of the evolution of infections and immune repertoire over time. There is a crucial role for mathematical modeling in balancing the benefits of reducing transmission with the costs of increasing susceptibility.

Two major concerns in malaria control are the severity of disease in infected individuals and their infectivity to mosquito vectors. In the absence of data on severity of infections and gametocyte counts, we have not specified these variables in the model, since the main goal was to simulate the dynamics of the PfEMP1 immune repertoire. However, the described results are likely to have implications on the patterns of disease severity and infectivity.

This model has not incorporated any degree of heterogeneity in host susceptibility, in the exposure to the parasite and the degree of treatment with antimalarials. It is known that individuals with certain genetic traits, apart from the sickle-cell trait, are less susceptible to develop blood-stage parasitemia [32]–[33]. Previously reported mathematical models of malaria have incorporated heterogeneities in exposure by including age-dependence [34], groups of high and low susceptibility/exposure [29], distributions of the duration of infection [35] and the effect of antimalarials [36]–[38]. Not incorporating these effects in the transmission models may compromise the accuracy of parameter estimates, and there is always room for model refinement. However, in the current study there was no need, when fitting the data, to incorporate age-dependent force of infection and clearance rates (age was not a significant factor associated with parasitemia in 2005 as assessed by the Kruskall-Wallis test, *p*  =  0.36), in coherence with a recent study [39].

Focusing on the dynamics of PfEMP1 immunity, apart from the fact that multiple events of exposure to the parasite are required to seroconvert to a given antigen, the fact that seroconversion to several variants per event was a more appropriate description to the given immunity is in agreement with previous models [18]; [40]. Despite the fact that the data available from the 2005 survey gives better support for the model where up to 6 variants are acquired in a single infection, it would be interesting to confront these predictions with follow-up data from the island. More mechanistic descriptions would require understanding of the intervals between parasitemia peaks in the field and the relative dominance of variants and acquisition of immunity to non-dominant variants. The incorporation of these factors may help to better understand the relationship between antigenic switching, intra-host competition and the possibility of acquisition of immunity to several variants per event.

It is also important to state that only intraclonal variability is being considered here, since almost all the recombinant proteins used for antibody detection were obtained from the 3D7 strain of *P. falciparum*. This strain is from unknown origin and might not be circulating anymore in the field [13]; [41]. Furthermore, the possibility of detecting antibodies against the listed recombinant proteins relies on the possibility of cross-reactivity of the antibodies of the individuals against domains of proteins of different clones or the degree of similarity between the variants expressed in the field and the ones expressed by the 3D7 isolate [42]. Another approach that would give a more informative view of the immunity against the strains present in the field would be a prior population genetics characterization of the *P. falciparum* strains in circulation and a measure of antibody responses against proteins they express. This approach has been used with success for characterization of antibody responses against PfEMP1 domains [41] and would allow to better characterize evolution and diversity of *var* genes in nature.

In this work, to define seropositivity we used a cut-off from responses of non-immune individuals, in agreement with previous models for seroconversion to a single antigen [17]. The use of a cut-off defined in this fashion is arbitrary and its use excludes the information about the magnitude of the immune response of each individual. The use of stochastic modeling would provide a more convenient framework to include individual variability on the levels of antibodies against each variant. With this information, it would be possible to characterize the variants in terms of immunogenicity and possibly to infer which variants are more commonly presented to the host immune system. As implemented here, the simulations refer to a counting process and do not consider which variants are present through time. Since there is evolution of the *var* genes under several factors including immune pressure and the composition of the susceptible pool in the population [43], despite the fact that the number of acquired variants in a person may be the same before the intervention and after a re-emergence, the risk of disease may not be the same because the variants may have changed. For a more detailed description of the framework of evolution and immune selective pressure on the *var* genes at the population level, a longitudinal framework for data acquisition would be more appropriate in order to know the immune and infectious history of the individuals.

Shortly, based on malaria epidemiology in a West African island, we have developed mathematical models to characterize and simulate infection and immunity scenarios for variant surface antigens of *P. falciparum*. These models and the proposed extensions may be applied to other settings and guide health policies by predicting the impact of current and prospective efforts for malaria elimination.

## Supporting Information

Figure S1 PfEMP1 model (7) with unitary seroconversion step and age data in 2005. True proportions per age group are represented by black circles. Gray curves: Estimated proportions by age for each category. Top: Individuals with no immunity. Middle: Individuals with immunity to 1–10 variants. Bottom: Individuals with immunity to more than 11 variants.
(TIF)

Figure S2 Equilibrium antibody repertoires assuming unitary seroconversion step. Mean number of PfEMP1 variants by age. Black circles: true means per age group in 2005. Black curve: model (7) fit to 2005 data. Dark gray: projection for 2008 (2.5 years after the beginning of the intervention). Light gray: projection for 2010 (5 years after the beginning of the intervention).
(TIF)

Figure S3 PfEMP1 variants averaged over the population assuming unitary seroconversion step. Curves generated by model (7). Black: transmission coefficient is reduced to half of its initial value, until the end of the simulation. Gray: transmission coefficient is reduced to half of its initial value, and relapses back to the initial value in 2011 (6 years after the intervention started).
(TIF)

Table S1 PfEMP1 variants and epitopes evaluated in this study.
(PDF)

Table S2 Parameter estimates for the PfEMP1 model (7) with unitary seroconversion step and 95% credible intervals.
(PDF)

Text S1 PfEMP1 model with 26 variants (*n*  =  26) and initial seroconversion step of 6 variants (*n*_0_  =  6).
(PDF)
