# Supplementary figures and images for: Modeling Malaria Infection and Immunity against Variant Surface Antigens in Príncipe Island, West Africa
Source: PLoS One. 2014 Feb 10;9(2):e88110. doi: 10.1371/journal.pone.0088110 (PMC3919732; doi:10.1371/journal.pone.0088110)

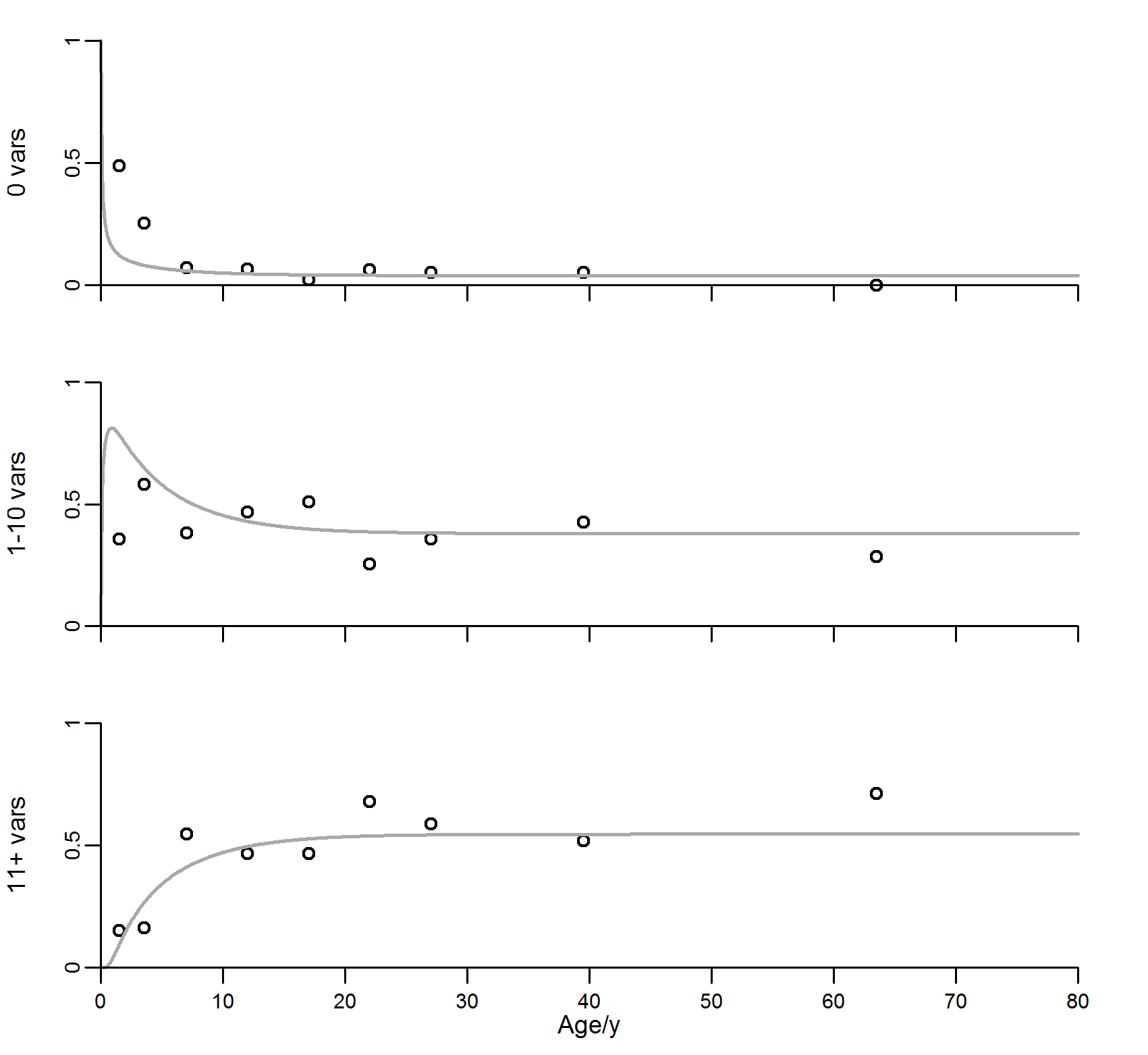

Supplement: Figure S1 — PfEMP1 model (7) with unitary seroconversion step and age data in 2005. True proportions per age group are represented by black circles. Gray curves: Estimated proportions by age for each category. Top: Individuals with no immunity. Middle: Individuals with immunity to 1–10 variants. Bottom: Individuals with immunity to more than 11 variants. (TIF) [file pone.0088110.s001.tif]

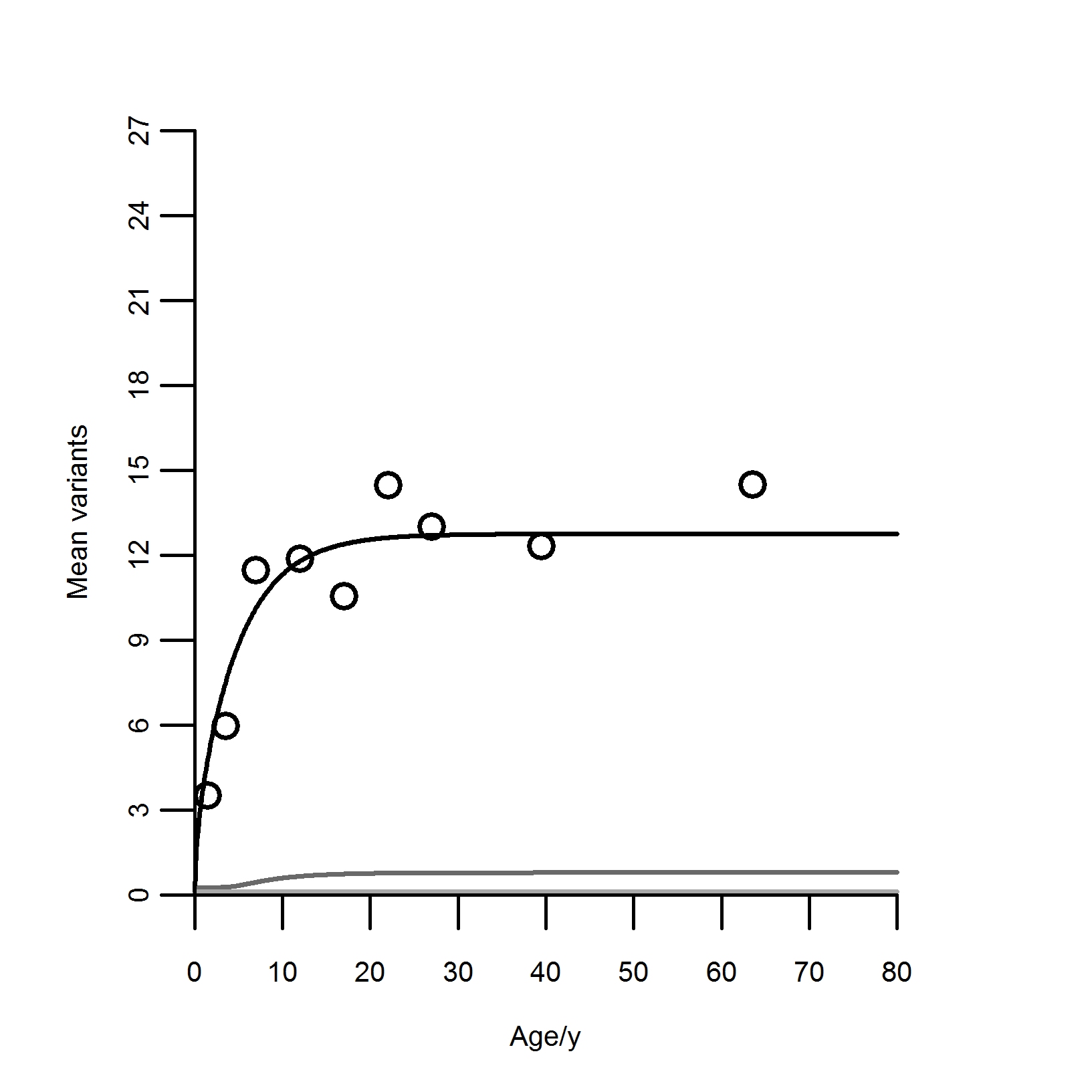

Supplement: Figure S2 — Equilibrium antibody repertoires assuming unitary seroconversion step. Mean number of PfEMP1 variants by age. Black circles: true means per age group in 2005. Black curve: model (7) fit to 2005 data. Dark gray: projection for 2008 (2.5 years after the beginning of the intervention). Light gray: projection for 2010 (5 years after the beginning of the intervention). (TIF) [file pone.0088110.s002.tif]

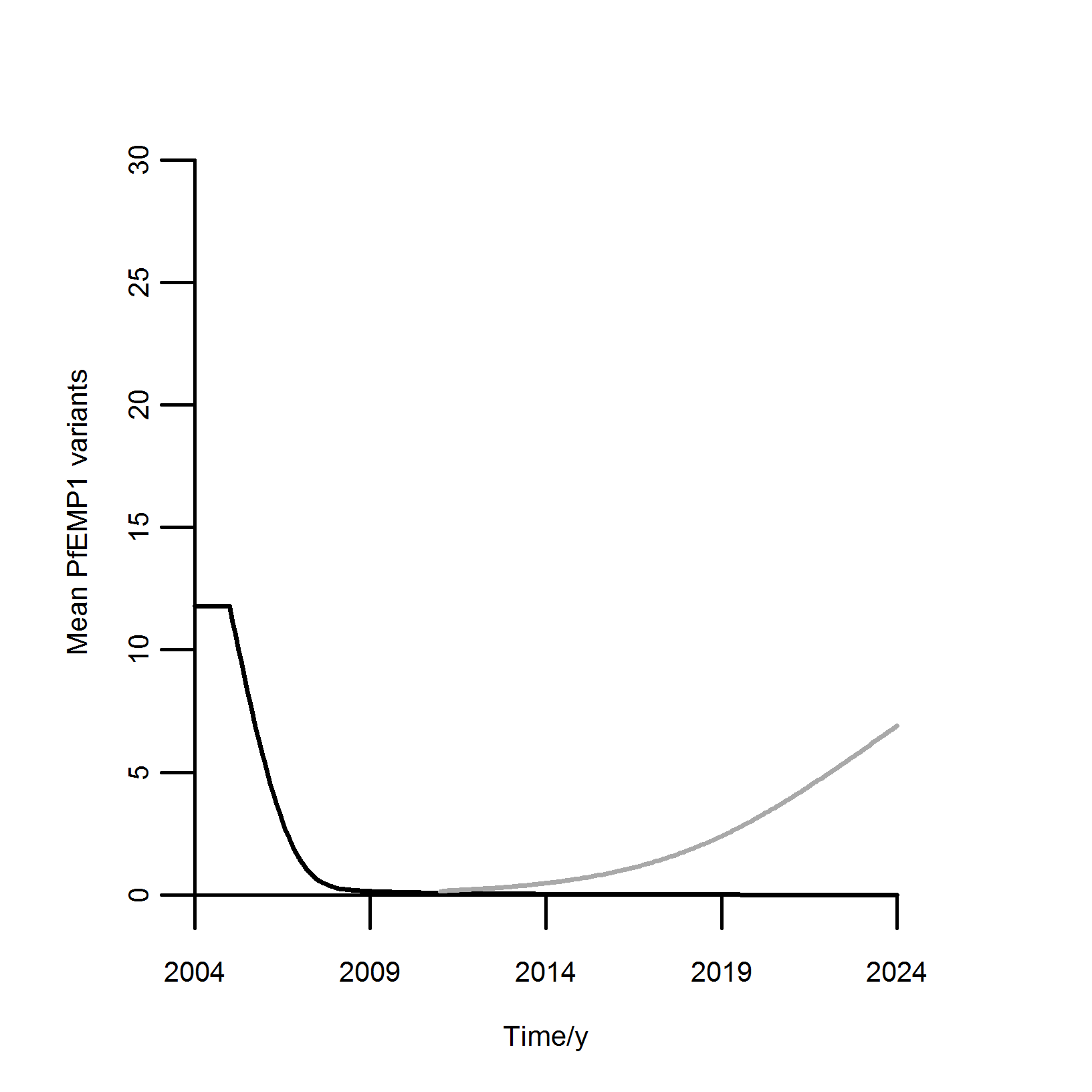

Supplement: Figure S3 — PfEMP1 variants averaged over the population assuming unitary seroconversion step. Curves generated by model (7). Black: transmission coefficient is reduced to half of its initial value, until the end of the simulation. Gray: transmission coefficient is reduced to half of its initial value, and relapses back to the initial value in 2011 (6 years after the intervention started). (TIF) [file pone.0088110.s003.tif]
