# Supplementary material for: Modeling Malaria Infection and Immunity against Variant Surface Antigens in Príncipe Island, West Africa
Source: PLoS One. 2014 Feb 10;9(2):e88110. doi: 10.1371/journal.pone.0088110 (PMC3919732; doi:10.1371/journal.pone.0088110)
Supplement: Table S1 — PfEMP1 variants and epitopes evaluated in this study. (PDF) [file pone.0088110.s004.pdf]

Table S1. PfEMP1 variants and epitopes evaluated in this study.

UID	PfEMP1	Genome	Domain Class	PfEMP1 group	Domain cassete
GK2_39	PF11_0521	3D7	CIDRα1.4	A	13
2324_63	PFD0020c	3D7	DBLγ6	A	8
2122_77	PFD0020c	3D7	DBLβ12	A	8
3028_48	PFI1820w	3D7	DBLα1.3-DBLε8	A	3
127128_76	PFE1640w	3D7	DBLβ1	A	1
GK26_59	PFL2665c	3D7	CIDRα2.3	C	Type 1
199200_90	IT4_var2	3D7	CIDRδ1	A	16
GK28_57	MAL6P1.1	3D7	CIDRα4	B	Type 1
GK23_60	PFL0005w	3D7	CIDRα2.2	B	Type 1
GK25_41	PFC0005w	3D7	CIDRα2.4	B	Type 1
129130_67	PFE1640w	3D7	DBLγ15	A	1
VAR5CIDR_37	PF11_0008	3D7	CIDRβ4	A	5
155156_65	MAL6P1.316	3D7	DBLζ3	BA	12
189190_51	IT4var21	FCR3	CIDRα3.1	B	Type 1
167168_61	HB3var22	HB3	DBLε2	BA	7
8788_52	PFD0005w	3D7	DBLδ1	B	None
SM1112_58	PFL1955w	3D7	CIDRα3.4	B	19
GK17_40	PFD0005w	3D7	CIDRα2.10	B	Type 1
6970_73	MAL6P1.4	3D7	DBLε2	BA	7
5556_43	PFL0020w	3D7	DBLε4	BA	6
E22-3_55	PF11_0008	3D7	DBLδ5	A	5
D20-24_42	PF11_0008	3D7	DBLγ12	A	5
179180a_46	PFD0615c	3D7	DBLδ1	C	Type 1
733_92	PF13_0003	3D7	CIDRδ1	A	16
660C_68	PFD1235w	3D7	DBLγ13	A	None
171174_34	MAL6P1.316	3D7	DBLγ4	BA	6/8
5354_64	PFL0020w	3D7	DBLζ5	BA	6
6768_89	MAL6P1.4	3D7	CIDRγ1	BA	43
6566_36	MAL6P1.4	3D7	DBLδ4	BA	None
6162_69	MAL6P1.4	3D7	DBLβ5	BA	14
GK37_49	PF08_0103	3D7	CIDRα2.2	B	Type 1
1516_75	PF08_0141	3D7	DBLζ5	A	6
1112_74	PF08_0141	3D7	DBLβ6	A	None
1314_66	PF08_0141	3D7	DBLγ14	A	6
101102_33	PF07_0049	3D7	DBLδ1	C	Type 1
2928_85	MAL6P1.314	3D7	VAR3/type3	A	3
7374_38	MAL6P1.4	3D7	DBLε3	BA	7
6364_84	MAL6P1.4	3D7	DBLγ13	BA	None
GK34_50	PFL1955w	3D7	CIDRα3.4	B	19
7172_72	MAL6P1.4	3D7	DBLε7	BA	7
4344_53	PF08_0140	3D7	CIDRα1.6	BA	None
3738_56	PF08_0140	3D7	DBLβ12	BA	8
2728_62	PFA0015c	3D7	DBLα1.3-DBLε8	A	3
P310_54	PFD1235w	3D7	CIDRα1.6	A	4
P308_47	PFD1235w	3D7	DBLγ13	A	none
