# Supplementary material for: Modeling Malaria Infection and Immunity against Variant Surface Antigens in Príncipe Island, West Africa
Source: PLoS One. 2014 Feb 10;9(2):e88110. doi: 10.1371/journal.pone.0088110 (PMC3919732; doi:10.1371/journal.pone.0088110)
Supplement: Table S2 — Parameter estimates for the PfEMP1 model (7) with unitary seroconversion step and 95% credible intervals. (PDF) [file pone.0088110.s005.pdf]

**Table S2. Parameter estimates for the PfEMP1 model (7) with unitary seroconversion step and 95% credible intervals.**

| Parameter | Mean estimate | Median estimate | 95% CI |
|---|---|---|---|
| λ | 14.738 | 14.4349 | 10.5549-20.2728 |
| ρ | 14.7999 | 14.4915 | 10.5487-20.4815 |
