# Supplementary material for: Modeling Malaria Infection and Immunity against Variant Surface Antigens in Príncipe Island, West Africa
Source: PLoS One. 2014 Feb 10;9(2):e88110. doi: 10.1371/journal.pone.0088110 (PMC3919732; doi:10.1371/journal.pone.0088110)
Supplement: Text S1 — PfEMP1 model with 26 variants (n = 26) and initial seroconversion step of 6 variants (n 0 = 6). (PDF) [file pone.0088110.s006.pdf]

Supporting Text S1 - PfEMP1 model with 26 variants (*n* = 26) and initial seroconversion step of 6 variants (*n*$_0$ = 6)

$$\frac{\partial P_0}{\partial t}+\frac{\partial P_0}{\partial a}=-(\lambda(t)+\mu)P_0+\rho P_1$$

$$\frac{\partial P_1}{\partial t}+\frac{\partial P_1}{\partial a}=-(\lambda(t)+\rho+\mu)P_1+\rho P_2$$

$$\frac{\partial P_2}{\partial t}+\frac{\partial P_2}{\partial a}=-(\lambda(t)+\rho+\mu)P_2+\rho P_3$$

$$\frac{\partial P_3}{\partial t}+\frac{\partial P_3}{\partial a}=-(\lambda(t)+\rho+\mu)P_3+\rho P_4$$

$$\frac{\partial P_4}{\partial t}+\frac{\partial P_4}{\partial a}=-(\lambda(t)+\rho+\mu)P_4+\rho P_5$$

$$\frac{\partial P_5}{\partial t}+\frac{\partial P_5}{\partial a}=-(\lambda(t)+\rho+\mu)P_5+\rho P_6$$

$$\frac{\partial P_6}{\partial t}+\frac{\partial P_6}{\partial a}=\lambda(t)P_0-(\lambda(t)+\rho+\mu)P_6+\rho P_7$$

$$\frac{\partial P_7}{\partial t}+\frac{\partial P_7}{\partial a}=\lambda(t)P_1-(\lambda(t)+\rho+\mu)P_7+\rho P_8$$

$$\frac{\partial P_8}{\partial t}+\frac{\partial P_8}{\partial a}=\lambda(t)P_2-(\lambda(t)+\rho+\mu)P_8+\rho P_9$$

$$\frac{\partial P_9}{\partial t}+\frac{\partial P_9}{\partial a}=\lambda(t)P_3-(\lambda(t)+\rho+\mu)P_9+\rho P_{10}$$

$$\frac{\partial P_{10}}{\partial t}+\frac{\partial P_{10}}{\partial a}=\lambda(t)(P_4+P_5)-(\lambda(t)+\rho+\mu)P_{10}+\rho P_{11}$$

$$\frac{\partial P_{11}}{\partial t}+\frac{\partial P_{11}}{\partial a}=\lambda(t)P_6-(\lambda(t)+\rho+\mu)P_{11}+\rho P_{12}$$

$$\frac{\partial P_{12}}{\partial t}+\frac{\partial P_{12}}{\partial a}=\lambda(t)P_7-(\lambda(t)+\rho+\mu)P_{12}+\rho P_{13}$$

$$\frac{\partial P_{13}}{\partial t}+\frac{\partial P_{13}}{\partial a}=\lambda(t)(P_8+P_9)-(\lambda(t)+\rho+\mu)P_{13}+\rho P_{14}$$

$$\frac{\partial P_{14}}{\partial t}+\frac{\partial P_{14}}{\partial a}=\lambda(t)P_{10}-\left(\lambda(t)+\rho+\mu\right)P_{14}+\rho P_{15}$$

$$\frac{\partial P_{15}}{\partial t}+\frac{\partial P_{15}}{\partial a}=\lambda(t)P_{11}-\left(\lambda(t)+\rho+\mu\right)P_{15}+\rho P_{16}$$

$$\frac{\partial P_{16}}{\partial t}+\frac{\partial P_{16}}{\partial a}=\lambda(t)\left(P_{12}+P_{13}\right)-\left(\lambda(t)+\rho+\mu\right)P_{16}+\rho P_{17}$$

$$\frac{\partial P_{17}}{\partial t}+\frac{\partial P_{17}}{\partial a}=\lambda(t)P_{14}-\left(\lambda(t)+\rho+\mu\right)P_{17}+\rho P_{18}$$

$$\frac{\partial P_{18}}{\partial t}+\frac{\partial P_{18}}{\partial a}=\lambda(t)P_{15}-\left(\lambda(t)+\rho+\mu\right)P_{18}+\rho P_{19}$$

$$\frac{\partial P_{19}}{\partial t}+\frac{\partial P_{19}}{\partial a}=\lambda(t)P_{16}-\left(\lambda(t)+\rho+\mu\right)P_{19}+\rho P_{20}$$

$$\frac{\partial P_{20}}{\partial t}+\frac{\partial P_{20}}{\partial a}=\lambda(t)\left(P_{17}+P_{18}\right)-\left(\lambda(t)+\rho+\mu\right)P_{20}+\rho P_{21}$$

$$\frac{\partial P_{21}}{\partial t}+\frac{\partial P_{21}}{\partial a}=\lambda(t)P_{19}-\left(\lambda(t)+\rho+\mu\right)P_{21}+\rho P_{22}$$

$$\frac{\partial P_{22}}{\partial t}+\frac{\partial P_{22}}{\partial a}=\lambda(t)P_{20}-\left(\lambda(t)+\rho+\mu\right)P_{22}+\rho P_{23}$$

$$\frac{\partial P_{23}}{\partial t}+\frac{\partial P_{23}}{\partial a}=\lambda(t)\left(P_{21}+P_{22}\right)-\left(\lambda(t)+\rho+\mu\right)P_{23}+\rho P_{24}$$

$$\frac{\partial P_{24}}{\partial t}+\frac{\partial P_{24}}{\partial a}=\lambda(t)P_{23}-\left(\lambda(t)+\rho+\mu\right)P_{24}+\rho P_{25}$$

$$\frac{\partial P_{25}}{\partial t}+\frac{\partial P_{25}}{\partial a}=\lambda(t)P_{24}-\left(\lambda(t)+\rho+\mu\right)P_{25}+\rho P_{26}$$

$$\frac{\partial P_{26}}{\partial t}+\frac{\partial P_{26}}{\partial a}=\lambda(t)P_{25}-\left(\rho+\mu\right)P_{26}$$

where P_i represents the proportion of individuals seropositive to i variants, $\lambda(t)$ is the time-dependent seroconversion rate, ρ is the constant seroreversion rate, and μ is mortality rate. The boundary conditions for the system at age a = 0 are $P_0(t,0)=\mu$ and $P_i(t,0)=0$, for $i \in \{1,\ldots,n\}$.
